# Dataset on insightful bio-evaluation of 2-(quinoline-4-yloxy)acetamide analogues as potential anti-*Mycobacterium tuberculosis* catalase-peroxidase agents via *in silico* mechanisms

**DOI:** 10.1016/j.dib.2021.107441

**Published:** 2021-10-01

**Authors:** Abel Kolawole Oyebamiji, Olubunmi Modupe Josiah, Sunday Adewale Akintelu, Moriam Dasola Adeoye, Babatunde Olasupo Sabitu, Dayo Felix Latona, Akintomiwa O. Esan, Emmanuel Ayodele Soetan, Banjo Semire

**Affiliations:** a Department of Pure and Applied Chemistry, Computational Chemistry Research Laboratory, Ladoke Akintola University of Technology, P.M.B. 4000, Ogbomoso, Oyo State, Nigeria; b Department of Basic Sciences, Adeleke University, Ede, Osun State, Nigeria; c School of Chemistry and Chemical Engineering, Beijing Institute of Technology, Beijing, China; d Department of Chemical Sciences, Fountain University, Osogbo, Nigeria; e National Agency for Food and Drug Administration and Control (NAFDAC), Abuja, Nigeria; f Department of Pure and Applied Chemistry, Osun State University, Osogbo, Nigeria; g School of Chemical Sciences, Universiti Sains Malaysia, Penang, Malaysia; h Department of Pharmacology, College of Medicine, Bowen University, Iwo, Osun State, Nigeria

**Keywords:** 2-(quinoline-4-yloxy)acetamide, Tuberculosis, QSAR, DFT, Docking, ADMET

## Abstract

The continuous havoc wrecked by tuberculosis among humans worldwide remains colossal. In this work, twenty-one (21) 2-(quinoline-4-yloxy)acetamide analogues were observed against *Mycobacterium tuberculosis* catalase-peroxidase (This enzyme shields bacteria from poisonous drug-like molecules) (PDB ID: 1sj2) using density functional theory method, QSAR study using material studio software and docking method via PyMol, AutoDock Tool, AutoDock Vina and Discovery studio 2017 as well as ADMET study via admetSAR2. Twelve descriptors were obtained from the optimized compounds which were used to develop valid QSAR model. More so, the binding affinity between 2-(quinoline-4-yloxy)acetamide analogues and *Mycobacterium tuberculosis* catalase-peroxidase (PDB ID: 1sj2) via docking method were reported. ADMET properties of some selected compounds were also examined.

## Specification Table

**Table:** 

Subject	Bioinformatics
Specific subject area	Drug Discovery
Type of data	Table Figure Statistical data Quantitative structural activity relationship (QSAR) model
How data were Acquired	Spartan’14, PaDEL-Descriptors, Data_pretreatment_train_test 1.0, Dataset division GUI v1.2_9, Pymol 1.7.4.4, Autodock_1.5.6; AutoVina, EduPyMOL-v1.7.4.4; Discovery studio 2019client
Data format	Raw Data
Parameters for data Collection	B3LYP, 6-31G*, Multiple linear regression (MLR), Genetic function approximation (GFA), EduPyMOL-v1.7.4.4-Win32, biovia2019.ds2019client, mgltools_win32_1.5.6 and Autodock vina
Description of data Collection	Twenty-one molecular compounds were theoretically investigated using density functional theory (DFT). The investigated compounds were divided into two sets (Training set and test set) and the descriptors from the training set were used to develop reliable QSAR model and test set was used to confirm it reliability via material studio software. All compounds were docked against *Mycobacterium tuberculosis* catalase-peroxidase via molecular docking method and ADMET properties of the compounds with highest binding affinity was examined before interpretation of result.
Data source location	Computational and Theoretical Chemistry Research Laboratory, Department of Basic Sciences, Adeleke University, Ede, Osun State, Nigeria
Data accessibility	All the data (experimental [1] and predicted) can be accessed in the data article
Related research article	A.F. Borsoi, J.D. Paz, B.L. Abbadi, F.S. Macchi, N. Sperotto, K. Pissinate, R.S. Rambo, A.S. Ramos, D. Machado, M. Viveiros, C.V. Bizarro, L.A. Basso, P. Machado. Design, synthesis, and evaluation of new 2-(quinoline-4-yloxy)acetamide-based antituberculosis agents. Eur J Med Chem 192 (2020) 112179.

## Value of the Data

• The calculated data (descriptors) from the optimized 2-(quinoline-4-yloxy)acetamide derivatives will help researchers to recognize descriptors which describe their inhibiting capacity.
• The selected descriptors from the optimized 2-(quinoline-4-yloxy)acetamide derivatives will also assist researchers to develop reliable and valid QSAR model with effective cytotoxicity.
• The calculated binding affinity will help scientists to locate 2-(quinoline-4-yloxy)acetamide based compound with utmost inhibiting ability against Mycobacterium tuberculosis catalase-peroxidase (PDB ID: 1sj2).
• The proposed drug-like molecules will assist researchers to have access to library of molecules with better inhibiting ability than the standard drug used in this work.

## 1 Data Description

Table 1 showed 2D structures of 2-(quinoline-4-yloxy)acetamide derivatives experimentally synthesised by Borsoi et al. [1] which was further converted to 3D and optimized using quantum chemical method via 6–31G^∗^ as basis set.

**Table 1:** 3D structures of 2-(quinoline-4-yloxy)acetamide derivatives.

	

	**R**
**1**	Ph
**2**	3-MeO-Ph
3*	3,5-(MeO)_2_-Ph
**4**	4-F-Ph
**5**	3-F-Ph
**6**	2-F-Ph
7*	3,4-(F)_2_-Ph
**8**	4-Cl-Ph
**9**	3-Cl-Ph
**10**	2-Cl-Ph
**11**	3,4-(Cl)_2_-Ph
**12**	2,3-(Cl)_2_-Ph
**13**	3-Cl-4-Br-Ph
**14**	4-Br-Ph
**15**	3-Br-Ph
**16**	4-F_3_C-Ph
**17**	3-F_3_C-Ph
**18**	4-O_2_N-Ph
**19**	4-i-Pr-Ph
**20**	4-t-Bu-Ph
**21**	2-Naphthyl
	**Proposed compound**
	
	**R_1_**
**P1**	CH_2_F
**P2**	CH_2_Cl
**P3**	CH_2_Br
**P4**	CH_3_

As shown in Table S1, twelve descriptors were obtained from the optimized 2-(quinoline-4-yloxy)acetamide derivatives and further screened for anti-tuberculosis activity. The descriptors obtained were highest occupied molecular orbital energy (E_HOMO_), lowest unoccupied molecular orbital energy (E_LUMO_), band gap, dipole moment, molecular weight, area, volume, ovality, lipophilicity (Log P), polarizability, hydrogen bond donor (HBD) and hydrogen bond acceptor (HBA) and the screened descriptors were also used for further analysis.

Table 2 displays the developed quantitative structure-activity relationship (QSAR) model using selected descriptors obtained from the optimized 2-(quinoline-4-yloxy)acetamide derivatives via series of software (Dataset Division GUI 1.2 software [2,3] and material studio software [4]). The descriptors involved in the developed QSAR model were E_HOMO_, Log P and HBA and the statistical factors considered for QSAR validation were adjusted squared correlation coefficient (Adj R^2^) (0.92), cross validation correlation coefficient (C.VR^2^) (0.89), P-Value (*P* < 0.0001) and F-Value (52.26). The predicted inhibition concentration (IC_50_) using the developed model were displayed in Table 3.

**Table 2:** Calculated QSAR model using selected descriptors from optimized 2-(quinoline-4-yloxy)acetamide derivatives.

Equation	R^2^	Adj. R^2^	C.VR^2^	*P*-value	*F*-value
IC_50_ = 59.690892769 (E_HOMO_)- 13.673062012(LogP) + 3.992788387(HBA) + 409.194825576	0.94	0.92	0.89	*P* < 0.0001	52.26

**Table 3:** Experimental and Predicted IC_50_ for 2-(quinoline-4-yloxy)acetamide derivatives.

	Experimental IC_50_	GFA
1	5.6	7.0
2	32.3	31.3
3*	29.5	18.7
4	16.8	19.3
5	16.8	18.7
6	19.2	18.1
7*	3.9	17.1
8	15.9	12.0
9*	7.9	12.0
10*	18.8	12.6
11	0.3	3.1
12*	28.7	4.3
13	1.6	-0.4
14	13.9	8.3
15*	13.9	7.7
16	7.2	8.7
17	7.2	7.5
18	30.8	31.1
19	1.9	3.9
20*	3.7	-1.8
21	7.6	8.1

⁎ Test Set

**Fig. 1:**
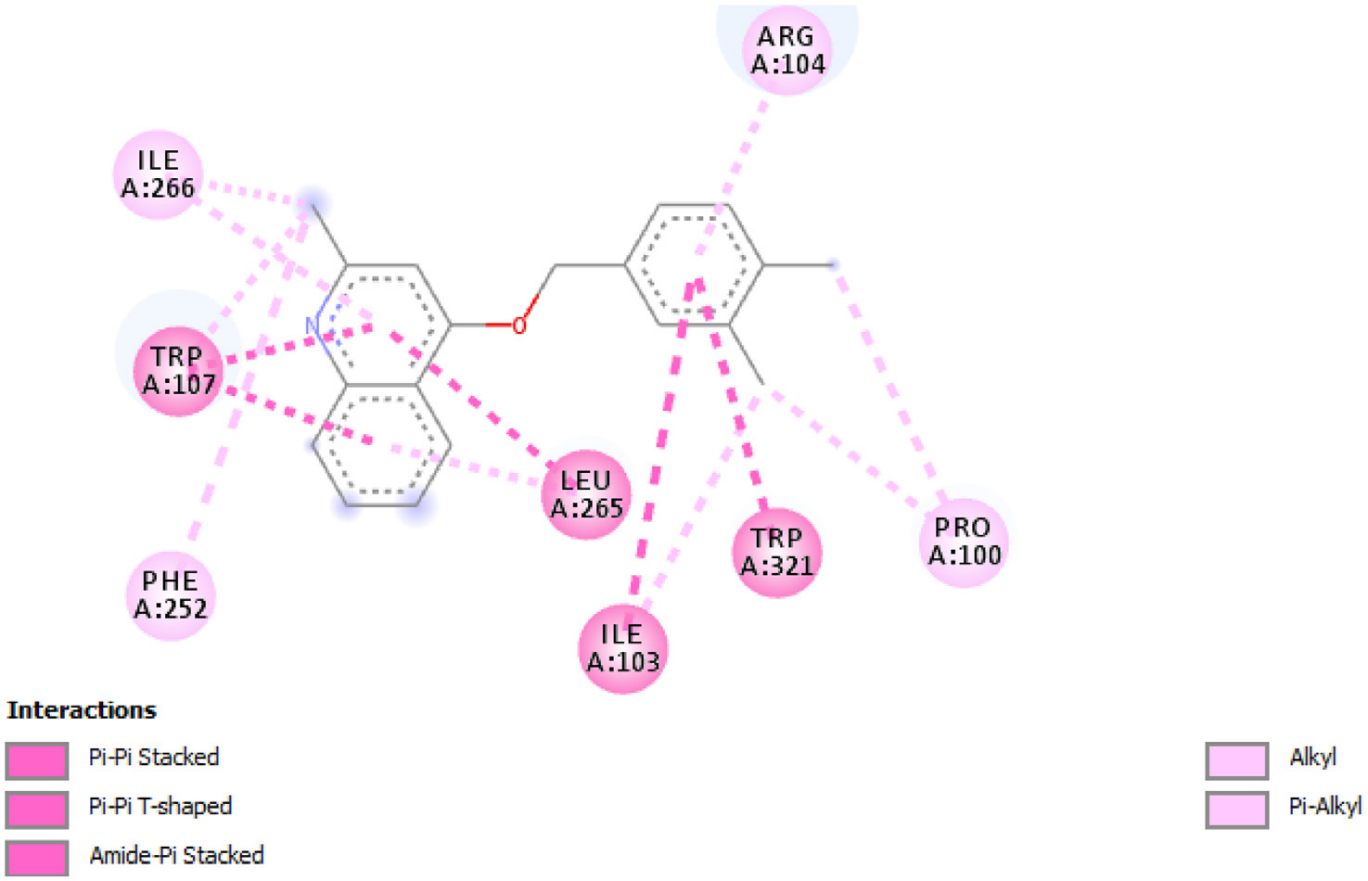
Residual interactions between compound **11** and *Mycobacterium tuberculosis* catalase-peroxidase (PDB ID: 1sj2).

**Fig. 2:**
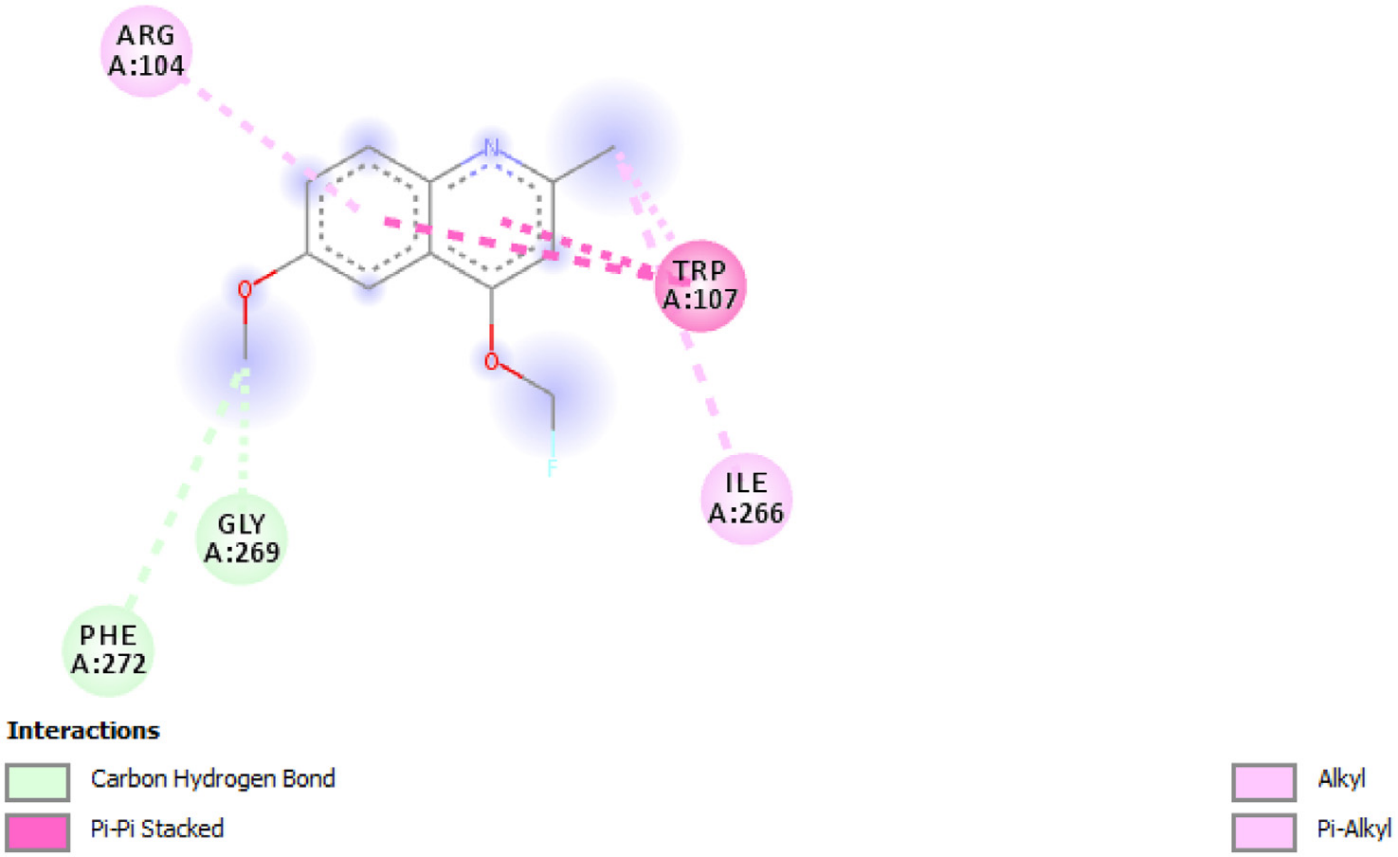
Residual interactions between compound **P1** and *Mycobacterium tuberculosis* catalase-peroxidase (PDB ID: 1sj2).

The binding affinity obtained between the optimized 2-(quinoline-4-yloxy)acetamide derivatives and *Mycobacterium tuberculosis* catalase-peroxidase (PDB ID: 1sj2) [5] were reported in Table 4. The calculated binding affinity for compound **1**–**21** were −10.1 kcal/mol, −8.2 kcal/mol, −7.8 kcal/mol, −8.2 kcal/mol, −10.9 kcal/mol, −8.5 kcal/mol, −11.3 kcal/mol, −8.4 kcal/mol, −10.5 kcal/mol, −7.9 kcal/mol, −11.4 kcal/mol, −7.5 kcal/mol, −8.5 kcal/mol, −7.5 kcal/mol, −10.4 kcal/mol, −9.2 kcal/mol, −8.9 kcal/mol, −7.9 kcal/mol, −7.4 kcal/mol, −8.0 kcal/mol and −11.2 kcal/mol and compared to the calculated binding affinity for the standard (Isoniazid) −6.0 kcal/mol. Four molecular compounds were also predicted and docked against *Mycobacterium tuberculosis* catalase-peroxidase (PDB ID: 1sj2) and their calculated binding affinity were compared to calculated binding affinity for Isoniazid (Table 4). The amino acid residues involved in the interaction between compound **11** as well as **P1** and *Mycobacterium tuberculosis* catalase-peroxidase were displayed in Figs. 1 and 2.

**Table 4:** Calculated scoring, residues and types of non-bonding interactions.

	Binding Affinity (kcal/mol)	Residues involved in the interactions	Types of Non-bonding interaction involved
**1.**	-10.1	Ile103, His270, Arg104, Trp107, Ile266, Leu265	Van der waals, Carbon Hydrogen Bond, Pi-Cation, Pi-Pi Stacked, Pi-Pi T-shaped, Amide-Pi Stacked, Alkyl, Pi-Alkyl
**2.**	−8.2	Ala478, Leu514,Arg595, Arg640, Asp511	Conventional Hydrogen bond, Pi-Anion, Alkyl, Pi-Alkyl
**3.**	-7.8	Arg595, Asp511, Arg640, Leu514	Pi-Anion, Alkyl, Pi-Alkyl
**4.**	-8.2	Arg640, Lys639, Ser474, Leu514, Ala478	Conventional Hydrogen bond, Halogen (Fluorine), Pi-Alkyl
**5.**	-10.9	Leu265, Trp107, Ile266, Phe252, Trp321, Ile103, Gly269, Pro100, Arg104	Halogen (Fluorine), Alkyl, Pi-Alkyl, Amide-Pi Stacked, Pi-Pi T-shaped, Pi-Pi Stacked
**6.**	-8.5	Ala478, Arg595, Leu514, Lys639, Asp509, Arg640	Conventional Hydrogen bond, Halogen (Fluorine), Alkyl, Pi-Alkyl
**7.**	-11.3	Ile266, Trp107, Phe252, Leu265, Ile103, Gly269, Gly273, Pro100, Phe272, Arg104	Conventional Hydrogen bond, Halogen (Fluorine), Pi-Pi Stacked, Amide-Pi Stacked, Alkyl, Pi-Alkyl
**8.**	-8.4	Leu514, Arg595, Arg640, Ala478	Conventional Hydrogen bond, Alkyl, Pi-Alkyl
**9.**	-10.5	Trp321, His270, Arg104, Ile266, Phe252, Trp107, Leu265	Conventional Hydrogen bond, Pi-Cation, Pi-Sigma, Pi-Pi Stacked, Pi-Pi T-shaped, Amide-Pi Stacked, Alkyl, Pi-Alkyl
**10.**	-7.9	Lys613, Ala591, Asp612, His116, Lys590, Leu616, Pro603	Conventional Hydrogen bond, Pi-Cation, Pi-Anion, Alkyl, Pi-Alkyl
**11.**	-11.4	Ile266, Trp107, Phe252, Ile103, Leu265, Trp321, Pro100, Arg104	Pi-Pi Stacked, Pi-Pi T-shaped, Amide-Pi Stacked, Alkyl, Pi-Alkyl
**12.**	-7.5	Arg640, Ser474, Asp513, Lys639	Conventional Hydrogen bond, Carbon Hydrogen bond, Pi-Donor Hydrogen Bond, Alkyl, Pi-Alkyl
**13.**	-8.5	Arg59, Tyr63	Conventional Hydrogen bond, Pi-Alkyl
**14.**	-7.5	Asp194, Gly120, Trp198, Asp194	Pi-Anion, Amide-Pi Stacked, Pi-Alkyl, Pi-Anion,
**15.**	-10.4	Trp107, Leu265, Trp412, Leu415, His270, Arg104, Trp321, Ile103, Ile266	Carbon Hydrogen Bond, Pi-Pi Stacked, Pi-Pi T-shaped, Amide-Pi Stacked, Alkyl, Pi-Alkyl
**16.**	-9.2	Thr475, Leu514, Ala478, Lys639, Ile497, Val517, Val507, Ser474, Arg640, Asp513	Van der waals, Carbon Hydrogen Bond, Halogen(Fluorine), Amide-Pi Stacked, Alkyl, Pi-Alkyl
**17.**	-8.9	Ala478, Arg595, Leu514, Tyr638, Lys639, Asp511, Asp509, Arg640	Conventional Hydrogen bond, Halogen(Fluorine), Pi-Anion, Alkyl, Pi-Alkyl
**18.**	-7.9	Leu616, Ala591, Asp612, Lys613, Lys590, His116, Pro219	Conventional Hydrogen bond, Carbon Hydrogen Bond, Pi-Cation, Pi-Anion, Alkyl, Pi-Alkyl
**19.**	-7.4	Val196, Gly120, Met126, Asp194, Trp198, Arg119	Pi-Anion, Pi-Donor Hydrogen Bond, Amide-Pi Stacked, Alkyl, Pi-Alkyl
**20.**	-8.0	Leu514, Arg595, Ala478, Arg640, Tyr638	Conventional Hydrogen bond, Alkyl, Pi-Alkyl
**21.**	-11.2	Trp107, Ile103, His270, Arg104, His108	Conventional Hydrogen bond, Pi-Cation, Pi-Sigma, Pi-Pi T-shaped, Amide-Pi Stacked, Pi-Alkyl
**INH**	-6.0	-	-

**Predicted Compounds**			

**P1**	-8.2	Arg104, Gly269, Phe272, Trp-107, Ile266	Carbon Hydrogen Bond, Pi-Pi Stacked, Alkyl, Pi-Alkyl
**P2**	-7.6	Lys693, Leu514, Ile497, Ala478, Arg595, Asn508	Conventional Hydrogen Bond, Carbon Hydrogen Bond, Alkyl, Pi-Alkyl
**P3**	-7.7	Leu265, Trp107, Gly269, Leu415, Trp412, His270, Arg104	Conventional Hydrogen Bond, Pi-Sigma, Pi-Pi Stacked, Alkyl, Pi-Alkyl
**P4**	-7.7	Arg104, Pro100, Gly269, Trp107, Phe252, Ile266	Carbon Hydrogen Bond, Pi-Pi Stacked, Alkyl, Pi-Alkyl

INH= Isoniazid

Table 5 shows the Lipinski rule of five for compounds with highest calculated binding affinity (Compound **11** and **P1** (from the proposed compounds). The calculated factors considered for the Lipinski rule of five were molecular weight ≤ 500 amu, AlogP ≤ 5, H-bond acceptor ≤ 10, h-bond donor≤ 5, rotatable bonds≤5. Also, the selected compounds (Compound 11 and P1) were subjected to adsorption, distribution, metabolism, excretion and toxicity analysis (ADMET) using admetsar 2 server (S2).

**Table 5:** Lipinski Rule of Five for compound **11, P1** and **INH.**

	Compound 11	Compound P1	INH
Molecular Weight	318.20	221.23	137.14
AlogP	5.43	2.86	-0.31
H-Bond Acceptor	2	3	3
H-Bond Donor	0	0	2
Rotatable bonds	3	3	1

## 2 Experimental Design, Materials and Methods

Twenty-one molecular compounds were optimized using density functional theory via Spartan 14 software [6]. In density functional theory method, three-parameter density functional which includes Becke's gradient exchange correction [7] and the Lee, Yang, Parr correlation functional. As reported by Semire *et al.,* (2017) [8], exactness of density functional theory (DFT) method is a function of the selected basis set; therefore, 6-31G* was used for optimization of the investigated drug-like molecules. The examined 2-(quinoline-4-yloxy)acetamide derivatives were:

4-(Benzyloxy)-6-methoxy-2-methylquinoline (**1**),
6-Methoxy-4-((3-methoxybenzyl)oxy)-2-methylquinoline (**2**),
4-((3,5-Dimethoxybenzyl)oxy)-6-methoxy-2-Methylquinoline (**3**),
4-((4-Fluorobenzyl)oxy)-6-methoxy-2-methylquinoline (**4**),
4-((3-Fluorobenzyl)oxy)-6-methoxy-2-methylquinoline (**5**),
4-((2-Fluorobenzyl)oxy)-6-methoxy-2-methylquinoline (**6**),
4-((3,4-Difluorobenzyl)oxy)-6-methoxy-2-methylquinoline (**7**),
4-((4-Chlorobenzyl)oxy)-6-methoxy-2-methylquinoline (**8**),
4-((3-Chlorobenzyl)oxy)-6-methoxy-2-methylquinoline (**9**),
4-((2-Chlorobenzyl)oxy)-6-methoxy-2-methylquinoline (**10**),
4-((3,4-Dichlorobenzyl)oxy)-6-methoxy-2-methylquinoline (**11**),
4-((2,3-Dichlorobenzyl)oxy)-6-methoxy-2-methylquinoline (**12**),
4-((4-Bromo-3-chlorobenzyl)oxy)-6-methoxy-2-Methylquinoline (**13**),
4-((4-Bromobenzyl)oxy)-6-methoxy-2-methylquinoline (**14**),
4-((3-Bromobenzyl)oxy)-6-methoxy-2-methylquinoline (**15**),
6-Methoxy-2-methyl-4-((4-(trifluoromethyl)benzyl)oxy)Quinolone (**16**),
6-Methoxy-2-methyl-4-((3-(trifluoromethyl)benzyl)oxy)Quinolone (**17**),
6-Methoxy-2-methyl-4-((4-nitrobenzyl)oxy)quinolone (**18**),
4-((4-Isopropylbenzyl)oxy)-6-methoxy-2-methylquinoline (**19**),
4-((4-(Tert-butyl)benzyl)oxy)-6-methoxy-2-methylquinoline (**20**),
6-Methoxy-2-methyl-4-(naphthalen-2-ylmethoxy)quinolone (**21**).

**Fig. 3:**
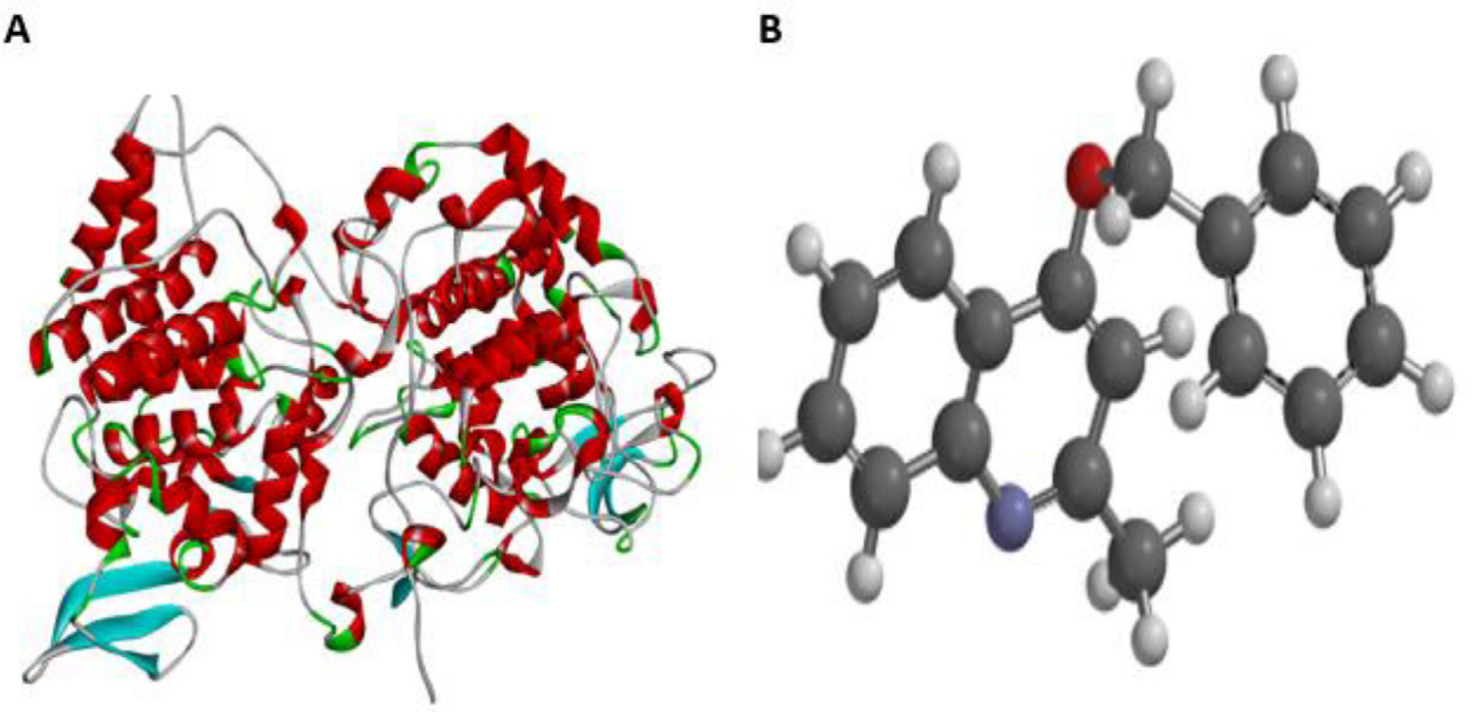
**A** 3D structure the target protein (PDB ID: 1sj2) and **B** 3D structure of the prepared 2-(quinoline-4-yloxy)acetamide derivatives.

The examined compounds were divided into two (training set and test set) and the descriptors from the training set compounds were used to developed reliable QSAR model whereas the test set was used to validate the predicting capacity of the developed QSAR model. In this work, four statistical factors (adjusted squared correlation coefficient (Adj.R^2^), cross validation squared correlation coefficient (C.VR^2^), F-value and P-value) were considered for QSAR validation. All the compounds investigated in this work were docked against *Mycobacterium tuberculosis* catalase-peroxidase (PDB ID: 1sj2) using series of software. The downloaded *Mycobacterium tuberculosis* catalase-peroxidase from protein data bank (www.rcsb.org) was subjected to PyMOL software so as to remove non-amino acid material before locating active site for docking calculation using autodock tool and autodock vina 1.1.2 respectively. The calculated grid box to identify the binding site for *Mycobacterium tuberculosis* catalase-peroxidase (PDB ID: 1sj2) was as follows: center (X = 39.493, Y = 5.682, Z = 43.68) and size (X = 24, Y = 32, Z = 116), the spacing was set to be 1.00Å and the exhaustiveness was set at default (8) (Fig. 3).

## Ethics Statement

Not applicable.

## CRediT authorship contribution statement

**Abel Kolawole Oyebamiji:** Conceptualization, Methodology, Writing – original draft. **Olubunmi Modupe Josiah:** Data curation. **Sunday Adewale Akintelu:** Software, Visualization. **Moriam Dasola Adeoye:** Data curation. **Babatunde Olasupo Sabitu:** Writing – review & editing. **Dayo Felix Latona:** Writing – review & editing. **Akintomiwa O. Esan:** Writing – review & editing. **Emmanuel Ayodele Soetan:** Software, Visualization. **Banjo Semire:** Supervision, Software, Validation.

## Declaration of Competing Interest

The authors declare that they have no known competing financial interests or personal relationships which have or could be perceived to have influenced the work reported in this article.

## Appendix Supplementary materials

Image, application 1
