# Supplementary material for: Dataset on insightful bio-evaluation of 2-(quinoline-4-yloxy)acetamide analogues as potential anti-Mycobacterium tuberculosis catalase-peroxidase agents via in silico mechanisms
Source: Data Brief. 2021 Oct 1;38:107441. doi: 10.1016/j.dib.2021.107441 (PMC8511800; doi:10.1016/j.dib.2021.107441)
Supplement: Supplementary file 1 [file mmc1.docx]

**Dataset on Insightful Bio-Evaluation of 2-(quinoline-4-yloxy)acetamide Analogues as Potential Anti-*Mycobacterium tuberculosis* Catalase-Peroxidase** **Agents Via *In silico* Mechanisms**

**OYEBAMIJI** Abel Kolawole^1,2^, **JOSIAH** Olubunmi Modupe^2^, **AKINTELU** Sunday Adewale^3^, **ADEOYE** Moriam Dasola^4^, **SABITU** Babatunde Olasupo^5^, **LATONA** Dayo Felix^6^, **ESAN** Akintomiwa O.^1,7^_,_ **SOETAN** Emmanuel Ayodele^8^ and **SEMIRE** Banjo^1^

^1^Computational Chemistry Research Laboratory, Department of Pure and Applied Chemistry, Ladoke Akintola University of Technology, P.M.B. 4000, Ogbomoso, Oyo State, Nigeria.

^2^Department of Basic Sciences, Adeleke University, Ede, Osun State, Nigeria

^3^School of Chemistry and Chemical Engineering, Beijing Institute of Technology, Beijing, China.

^4^Department of Chemical Sciences, Fountain University, Osogbo, Nigeria.

^5^National Agency for Food and Drug Administration and Control (NAFDAC), Abuja, Nigeria.

^6^Department of Pure and Applied Chemistry, Osun State University, Osogbo, Nigeria.

^7^School of Chemical Sciences, Universiti Sains Malaysia, Penang, Malaysia.

^8^Department of Pharmacology, College of Medicine, Bowen University, Iwo, Osun State.

*Email: [abeloyebamiji@gmail.com](mailto:abeloyebamiji@gmail.com), oyebamiji.abel@adelekeuniversity.edu.ng

**Table S1**: Calculated 3D Descriptors from optimized 2-(quinoline-4-yloxy)acetamide derivatives

|  | E_HOMO_ | E_LUMO_ | BG | DM | MW | AREA | VOL | OVA | LogP | POL | HBD | HBA |
| --- | --- | --- | --- | --- | --- | --- | --- | --- | --- | --- | --- | --- |
| 1 | -6.42 | -1.17 | 5.25 | 2.82 | 249.31 | 282.71 | 271.93 | 1.39 | 1.97 | 62.19 | 0 | 2 |
| 2 | -6.11 | -1.58 | 4.53 | 3.96 | 279.33 | 318.13 | 300.46 | 1.47 | 1.84 | 64.68 | 0 | 3 |
| 3* | -5.93 | -1.59 | 4.34 | 3.01 | 309.36 | 346.48 | 327.41 | 1.51 | 1.71 | 66.91 | 0 | 4 |
| 4 | -6.18 | -1.56 | 4.62 | 2.59 | 267.30 | 294.39 | 278.03 | 1.43 | 2.12 | 62.84 | 0 | 2 |
| 5 | -6.19 | -1.57 | 4.62 | 2.83 | 267.30 | 294.36 | 278.01 | 1.43 | 2.12 | 62.83 | 0 | 2 |
| 6 | -6.20 | -1.59 | 4.61 | 3.09 | 267.30 | 292.59 | 277.87 | 1.42 | 2.12 | 62.82 | 0 | 2 |
| 7* | -6.18 | -1.56 | 4.62 | 2.96 | 285.29 | 300.24 | 282.71 | 1.44 | 2.28 | 63.22 | 0 | 2 |
| 8 | -6.21 | -1.60 | 4.61 | 2.66 | 283.75 | 304.03 | 286.90 | 1.45 | 2.52 | 63.56 | 0 | 2 |
| 9* | -6.21 | -1.60 | 4.61 | 2.97 | 283.75 | 304.00 | 286.87 | 1.45 | 2.52 | 63.55 | 0 | 2 |
| 10* | -6.20 | -1.59 | 4.61 | 3.20 | 283.75 | 301.80 | 286.77 | 1.44 | 2.52 | 63.55 | 0 | 2 |
| 11 | -6.23 | -1.62 | 4.61 | 3.30 | 318.20 | 319.15 | 300.55 | 1.47 | 3.08 | 64.66 | 0 | 2 |
| 12* | -6.21 | -1.61 | 4.6 | 3.27 | 318.20 | 316.72 | 300.46 | 1.46 | 3.08 | 64.66 | 0 | 2 |
| 13 | -6.23 | -1.63 | 4.6 | 3.27 | 362.65 | 323.71 | 305.12 | 1.48 | 3.35 | 65.04 | 0 | 2 |
| 14 | -6.21 | -1.60 | 4.61 | 2.69 | 328.20 | 308.72 | 291.47 | 1.45 | 2.79 | 63.93 | 0 | 2 |
| 15* | -6.22 | -1.61 | 4.61 | 3.03 | 328.20 | 308.68 | 291.45 | 1.45 | 2.79 | 63.93 | 0 | 2 |
| 16 | -6.18 | -1.60 | 4.58 | 1.93 | 317.31 | 326.77 | 306.10 | 1.49 | 2.89 | 65.12 | 0 | 2 |
| 17 | -6.20 | -1.58 | 4.62 | 3.73 | 317.31 | 325.33 | 306.05 | 1.48 | 2.89 | 65.11 | 0 | 2 |
| 18 | -6.21 | -2.78 | 3.43 | 5.70 | 294.31 | 315.13 | 295.44 | 1.47 | 2.00 | 64.53 | 0 | 5 |
| 19 | -6.19 | -1.57 | 4.62 | 3.20 | 291.39 | 346.32 | 327.98 | 1.51 | 3.20 | 66.89 | 0 | 2 |
| 20* | -6.19 | -1.57 | 4.62 | 3.17 | 305.42 | 365.51 | 346.27 | 1.53 | 3.62 | 68.37 | 0 | 2 |
| 21 | -5.79 | -1.55 | 4.24 | 3.23 | 375.47 | 414.85 | 407.96 | 1.56 | 4.64 | 73.47 | 0 | 2 |

**Note:** E_HOMO_= Highest occupied molecular orbital energy; E_LUMO_= Lowest unoccupied molecular orbital energy; BG= Band Gap: DM= Dipole Moment; MW= Molecular weight; VOL= Volume; POL= Polarizability; HDB= Hydrogen bond donor; HBA= Hydrogen Bond Acceptor

Table S2: ADMET Properties for compound **11**, **P1** and **INH**

|  | Compound 11 | Compound P1 | INH |
| --- | --- | --- | --- |
|  | Probability | Probability | Probability |
| Ames mutagenesis | + | + | + |
| Acute Oral Toxicity (c) | III | III | III |
| Androgen receptor binding | + | + | - |
| Aromatase binding | + | + | - |
| Avian toxicity | - | - | - |
| Blood Brain Barrier | + | + | + |
| BRCP inhibitior | - | - | - |
| Biodegradation | - | - | - |
| BSEP inhibitior | + | - | - |
| Caco-2 | + | + | + |
| Carcinogenicity (binary) | - | - | - |
| Carcinogenicity (trinary) | Non-required | Non-required | Warning |
| crustacea aquatic toxicity | + | + | - |
| ssCYP1A2 inhibition | + | + | + |
| CYP2C19 inhibition | + | + | - |
| CYP2C9 inhibition | - | - | - |
| CYP2C9 substrate | - | - | - |
| CYP2D6 inhibition | - | - | - |
| CYP2D6 substrate | - | - | - |
| CYP3A4 inhibition | - | + | - |
| CYP3A4 substrate | + | - | - |
| CYP inhibitory promiscuity | + | + | - |
| Eye corrosion | - | - | - |
| Eye irritation | - | + | + |
| Estrogen receptor binding | + | + | - |
| Fish aquatic toxicity | + | - | - |
| Glucocorticoid receptor binding | + | - | - |
| Honey bee toxicity | - | + | - |
| Hepatotoxicity | + | + | + |
| Human either-a-go-go inhibition | + | + | - |
| Human Intestinal Absorption | + | + | + |
| Human oral bioavailability | + | + | + |
| MATE1 inhibitior | - | - | - |
| micronuclear | + | + | - |
| Acute Oral Toxicity | 1.943962574 | 1.344662309 | 1.167112 |
| OATP1B1 inhibitior | + | + | + |
| OATP1B3 inhibitior | + | + | + |
| OATP2B1 inhibitior | - | - | - |
| OCT1 inhibitior | + | + | - |
| OCT2 inhibitior | - | - | - |
| P-glycoprotein inhibitior | - | - | - |
| P-glycoprotein substrate | - | - | - |
| PPAR gamma | + | - | - |
| Plasma protein binding | 0.94824487 | 1.02630496 | 0.519366 |
| Subcellular localzation | Mitochondria | Mitochondria | Mitochondria |
| Tetrahymena pyriformis | 1.773569584 | 0.86533916 | -0.0492 |
| Thyroid receptor binding | + | + | - |
| UGT catelyzed | - | - | - |
| Water solubility | -4.205280467 | -3.147822323 | -0.05213 |
